# Nanoparticle Enhanced Antibody and DNA Biosensors for Sensitive Detection of *Salmonella*

**DOI:** 10.3390/ma11091541

**Published:** 2018-08-27

**Authors:** Sumeyra Savas, Aylin Ersoy, Yakup Gulmez, Selcuk Kilic, Belkis Levent, Zeynep Altintas

**Affiliations:** 1 National Research Institute of Electronics and Cryptology, The Scientific and Technological Research Council of Turkey (TUBITAK), Kocaeli 41470, Turkey; sumeyra.savas@tubitak.gov.tr (S.S.); aylin.ersoy@tubitak.gov.tr (A.E.); yakup.gulmez@tubitak.gov.tr (Y.G.); 2 Turkey Public Health General Headquarter, Ankara 06100, Turkey; selcuk.kilic@sbu.edu.tr (S.K.); belkislevent@hotmail.com (B.L.); 3 Institute of Chemistry, Technical University of Berlin, Straße des 17. Juni 124, Berlin 10623, Germany

**Keywords:** *Salmonella* spp., pathogen detection, antibody biosensor, DNA biosensor, microfluidic-based electrochemical sensor, nanoparticle enhanced bio-detection, infectious diseases

## Abstract

Bacteria-related pathogenic diseases are one of the major health problems throughout the world. *Salmonella* is a genus of rod-shaped Gram-negative enterobacteria of which more than 2600 serotypes have been identified. Infection with *Salmonella* can cause salmonellosis, a serious bacterial toxi-infection syndrome associated with gastroenteritis, and paralyphoid and typhoid fevers. Its rapid and sensitive detection is a key to the prevention of problems related to health. This paper describes the development of antibody and DNA sensors for *Salmonella* detection using a microfluidic-based electrochemical system. Commercial *Salmonella typhimurium* and *Salmonella typhimurium* from human stool samples were investigated using standard and nanomaterial-amplified antibody sensors. *S. typhimurium* could be detected down to 1 cfu mL^−1^. The specificity of immunoassay was tested by studying with non-specific bacteria including *E. coli* and *S. aureus* that revealed only 2.01% and 2.66% binding when compared to the target bacterium. On the other hand, the quantification of *Salmonella* DNA was investigated in a concentration range of 0.002–200 µM using the developed DNA biosensor that demonstrated very high specificity and sensitivity with a detection limit of 0.94 nM. Our custom-designed microfluidic sensor offers rapid, highly sensitive, and specific diagnostic assay approaches for pathogen detection.

## 1. Introduction

Foodborne diseases lead to diverse health problems worldwide [1,2]. The World Health Organization reported that *Salmonella typhimurium* and *Salmonella enteritidis* are the most common causes of foodborne illnesses all over the world. According to the reports of the European Food Safety Authority, human salmonellosis has resulted in three billion euros loss per year [3]. In some regions, more than 90% of *Salmonella* strains isolated from humans until 1970 was *S. typhimurium*, but now incidence of *S. enteritidis* is also gradually increasing, which has been the most frequently isolated serotype in the last 10 years [4]. The symptoms of *Salmonella* infection include abdominal pain, fever, nausea, vomiting, diarrhea, dehydration, weakness, and loss of appetite, and the symptoms normally appear 12–72 h after ingestion of contaminated foods or beverages [5].

There are several methods used for the detection of *Salmonella* serotypes, such as cultivation techniques [6], enzyme-linked immunosorbent assays (ELISAs) [7], polymerase chain reaction (PCR) methods [8,9,10], and biosensors [11]. The gold standard for detection of *Salmonella* serotypes are still conventional methods that are sensitive and inexpensive. However, these techniques require more than five days to obtain a result and often lack in providing good specificity and sensitivity. Additionally, the cultivation techniques are generally time consuming and the limit of detection for the analysis is insufficient. The use of biosensor technology is a strong alternative to the other techniques by offering highly sensitive, rapid, and easy-to-use bio-detection principles [12]. Today, biosensors are widely used for pathogen detection and are able to measure bacteria down to 1 cfu mL^−1^. This is particularly due to the significant impact of nanomaterials on the advancement of biosensors and biosensing principles [13,14,15]. Furthermore, microbial biosensors often require sample volumes in the microliter range and very short analysis time. 

Various transducer systems based on surface plasmon resonance (SPR) [16], quartz crystal microbalance (QCM) [17], and electrochemical sensing strategies [18] have been successfully employed for bacteria detection. Offering very high sensitivity and good detection capacity electrochemical sensors are among the widely used systems for *Salmonella* quantification [14,19,20]. Zhu et al. developed a multichannel electrochemical immunosensor for *Salmonella* detection by combining the rolling circle amplification with DNA-gold nanoparticles (AuNPs) probe [20]. Afonso et al. reported a disposable immunosensor for electrochemical detection of *Salmonella enterica* subsp. Enterica serovar Typhimurium LT2 using gold nanoparticles and magneto-immunoassay [14]. Sensitive amperometric detection of *Salmonella* was also reported [21,22,23,24]. Even though great progress has been demonstrated in the *Salmonella* detection, employing only one antibody in the biosensor design often results in insufficient sensitivity, when the sensor could hardly discriminate between two LPS samples from two different Gram-negative bacteria [25]. 

To overcome the selectivity problem, herein, we report antibody- and DNA-based biosensors for *Salmonella* detection using a fully-automated custom-designed microfluidic sensing device (MiSens) [18] that is composed of an electromechanical unit controlling the assay protocol via its integrated software (MiCont^TM^). Normal and gold-nanoparticle amplified sandwich assays were developed and used for the detection of commercial *Salmonella* samples and real samples from human stool. DNA biosensor was developed by capturing the surface DNA probe on the neutravidin (NA) immobilized sensor surface and then measuring the target *Salmonella* DNA based on the hybridization reaction that occurs between the target DNA and the surface probe. As the measurement system relies on the enzymatic reaction between horseradish peroxidase (HRP) and 3,3′,5,5′-tetramethylbenzidine (TMB), the detector antibody and the DNA detection probe were both labeled with HRP. We have demonstrated that the developed antibody and DNA biosensors are capable of measuring trace amounts of *Salmonella* and *Salmonella* DNA, respectively.

## 2. Materials and Methods 

### 2.1. Materials and Reagents

A monoclonal anti-*Salmonella* antibody was bought from BIO-RAD (Puchheim, Germany). Peroxidase-labeled goat anti-*Salmonella* secondary antibody (BacTrace^®^ Anti-*Salmonella* CSA-1 Antibody) and *Salmonella typhimurium* were purchased from SeraCare Life Sciences (Gaithersburg, MD, USA). *Staphylococcus aureus* and *E. coli* from human stool samples were obtained from the Public Health Agency (Ankara, Turkey) for cross-reactivity studies. 11-Mercaptoundecanoic acid (MUDA), phosphate-buffered saline tablets (PBS, 0.01 M phosphate buffer, 0.0027 M potassium chloride and 0.137 M sodium chloride, pH 7.4), N-hydroxysuccinimide (NHS), analytical grade ethanol, horseradish peroxidase (HRP), ethanolamine, and 3,3′,5,5′-tetramethylbenzidine (TMB) ready to use reagent with H_2_O_2_, were purchased from Sigma Aldrich (Poole, UK). The gold nanoparticles in 15 nm (for DNA assays) and 40 nm (for antibody assays) sizes were purchased from BBI International (Cardiff, UK). Ultrapure water (18 MΩ cm^−1^) produced by a Milli-Q water system was used for analyses (Millipore Corp., Tokyo, Japan). 1-Ethyl-3-(3-dimethylaminopropyl)-carbodiimide (EDC) and biotin were purchased from Thermo Fisher Scientific (Loughborough, UK). The oligonucleotide sequences (target sequence: 5′-ACCGACGGCGAGACCGACTTT-3′; surface probe: Biotin-5′-CTCACCAGGAGATTACAACATGG; detection probe: Biotin-3′-AGTGGCTAAAAGTCGGTCTC; control surface probe: Biotin-5′- CAATATTTGGCGTGAATGGGTCGGAAAACA) for DNA sensor development were obtained from Sentromer DNA Technologies LLC (Istanbul, Turkey).

### 2.2. Isolation of Salmonella Typhimurium from Human Stool Samples

Human stool sample was inoculated onto *Salmonella-Shigella* and xylose lysine deoxycholate agar mediums, and incubated at 37 °C overnight. Bacterial culture was identified by using standard biochemical tests (the use of glucose, citrate, and indole by bacteria, the production of gas and H_2_S upon lactose fermentation of bacteria, the determination of the mobility and the ability to split urea). The cultured colonies used glucose, citrate and indole confirmed the presence of *Salmonella* bacteria. The isolate was serogrouped and serotyped using polyvalent and monovalent *Salmonella* antisera (Statens Serum Institut, Copenhagen, Denmark) according to the Kauffmann-White scheme [26]. The colonies that produced H_2_S were considered as a pure colony based on their morphology. After the confirmation and serotyping, the strain was immediately frozen in tryptic soy broth with 16% glycerol at −80 °C.

### 2.3. Fully-Automated Microfluidic-Based Electrochemical Sensor with a New Chip Design

**Figure 1:**
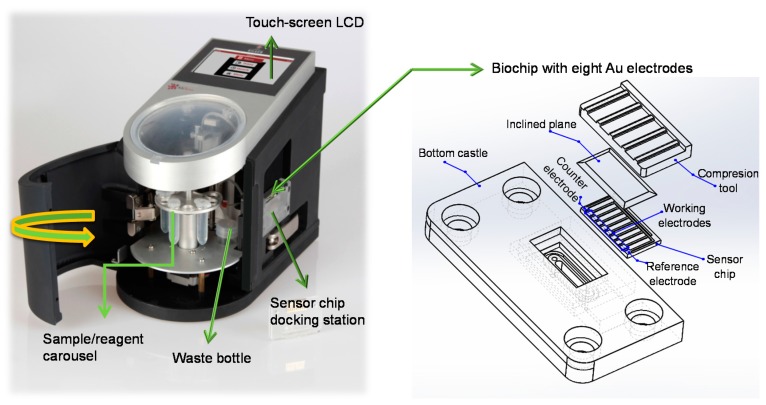
Illustration of MiSens electrochemical sensor and its new chip design.

A custom-designed fully-automated electrochemical sensor was used in this study to develop antibody- and DNA-based biosensors for *Salmonella* detection. The sensor fabrication and its developmental stages were fully described in our earlier studies [18,27,28]. Meanwhile, we have realized several drawbacks dealing with device’s electronics and mechanics. One of the main problems was electromagnetic noise in the signal. In order to decrease the noise level, two modifications were employed: (1) Increasing of the distance between the microfluidic pumps and the potentiometer. The microfluidic pump spread unwanted magnetic pulses by the pulse width modulation (PWM) mechanism; (2) Applying a coaxial cable structure instead of unscreened cable structure between the biosensor and potentiostat circuit. Herein, we designed and integrated a new chip (Figure 1) to improve the efficiency of the biosensing device and the reproducibility of the assays. The electrodes were designed on the glass slide (10 × 20 mm) using a fine metal mask, which was made of a laser-cut patterned stainless steel. Au was deposited on the wafer by means of an electron beam evaporator (Ebeam system Nanovak NVEB-600, Nanovak, Ankara, Turkey). Prior to the application of Au (200 nm), a 40 nm Ti layer was applied on to the wafer as an intermediary adhesive layer to enhance the adhesion between the glass slide and the Au. Each array contains eight working electrodes with a shared Au counter and quasi-reference electrodes. The experiments were carried out using the MiCont^TM^ software (TUBİTAK-BİLGEM, Kocaeli, Turkey) running on a wireless PC. The assay protocols were generated, saved, and used when required. 

### 2.4. Sensor Chip Cleaning and SAM Deposition

Prior to forming a self-assembled monolayer (SAM) on the sensor chip, the electrode surfaces were cleaned by employing nitrogen plasma [29,30]. A 2 mM concentration of MUDA was used to prepare the thiol solution in absolute ethanol for SAM deposition [18]. The sensor chips were immersed in the ethanolic solution for overnight followed by washing with ethanol and Milli-Q water, respectively. Later on the sensor chips were dried thoroughly under a stream of nitrogen gas, vacuum-packed, and stored at +4 °C till use.

### 2.5. Selection of HRP Concentration for Bioassays

Different concentrations of HRP were initially measured using MiSens device (TUBİTAK-BİLGEM, Kocaeli, Turkey) to determine the optimum HRP amount for bioassays. For this, six different concentrations of HRP were mixed with same amount of TMB and injected to the MUDA coated surfaces. These optimization experiments were repeated three times and the optimum HRP concentration was chosen based on the average sensor signals. 

### 2.6. Characterization of SAM Coated Sensor Chips Using AFM

The bare gold, the SAM coated and the antibody immobilized sensor chips were visualized by employing a Naio (Nanosurf AG, Liestal, Switzerland) atomic force microscope (AFM). Commercially available AFM probes (NCLR) from NanoWorld (NanoWorld AG, Liestal, Switzerland) were used for AFM measurements. The AFM analyses for the SAM-deposited surface and the antibody immobilization were carried out after having completed three cycles of buffer wash flow. The sensor surfaces during the DNA assays were also visualized using AFM. Hence, the NA immobilization, the capturing of DNA surface probe on the NA layer, and the target DNA binding were confirmed. The chips were undocked from the MiSens sensor after three cycles of buffer flow, dried using a gentle nitrogen stream, and then immediately placed in a glass Petri dish. All AFM measurements were performed at room temperature using the intermittent air mode. 

### 2.7. Development of the Antibody Biosensor

**Scheme 1:**
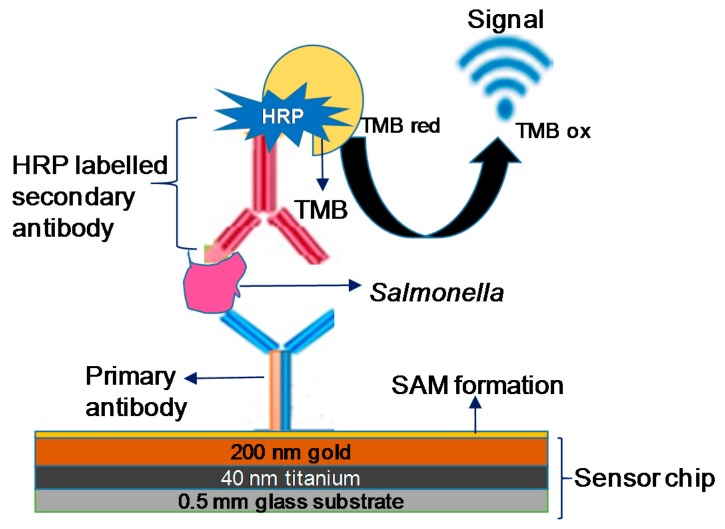
Principle of antibody sensor development for *Salmonella* detection using the standard assay.

**Scheme 2:**
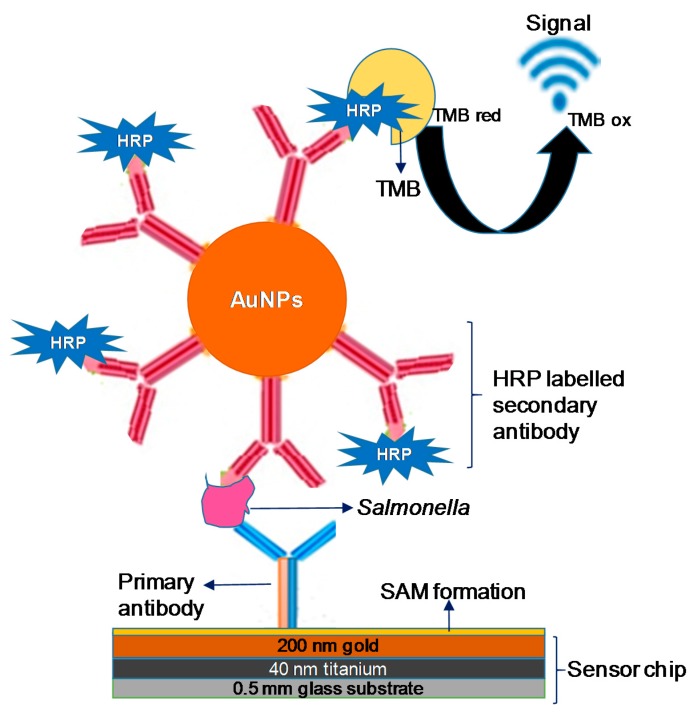
Principle of the gold nanoparticles enhanced immunosensor for *Salmonella* detection.

Two different antibody sensors were developed in this work by using a monoclonal primary antibody and a secondary polyclonal antibody for *Salmonella*. The primary antibody was used as a surface ligand to specifically capture *Salmonella* bacterium, whereas the polyclonal antibody was utilized as a detector antibody to increase the sensor signal. Two different bio-detection assays were established: a standard sandwich assay (Scheme 1) and a nanoparticle enhanced sensor assay (Scheme 2). In the latter case the polyclonal antibodies were initially conjugated with gold nanoparticles prior to the *Salmonella* detection assay. 

The running buffer used for immobilization was degassed buffered saline (PBS, pH 7.4) and this buffer continuously flowed over the Au sensor surfaces between the injections. The flow rate of the buffer/reagents for the assay was 50 μL min^−1^ unless written otherwise. For the assays, the SAM deposited sensor chip was initially inserted to the device and primed with the running buffer (PBS). The sensor surface was activated with a mixture of EDC (0.4 M) and NHS (0.1 M) in a 1:1 volume ratio by 4 min injection prior to the covalent immobilization of the primary antibody (50 μg mL^−1^, prepared in NaAc buffer, pH: 4.5) during the 4 min injection (50 μL min^−1^, 200 μL). The antibody-free areas of the sensing surface were then blocked using a 100 μg mL^−1^ BSA solution (50 μL min^−1^, 200 μL) and 1 M of ethanolamine (pH: 8.5, 50 μL min^−1^, 200 μL), respectively. Bio-detection capacity of the sensor was tested in the investigation range of 1–5.41 × 10^7^ cfu mL^−1^ using two different *Salmonella* sample sources: commercially available *S. typhimurium* and real *S. typhimurium* samples from human stool. The samples were prepared in a particular PBS buffer containing 200 μg mL^−1^ BSA, 0.5 M NaCl, 500 μg mL^−1^ dextran, and 0.5% Tween 20. Each sample was injected to the sensor surface (50 μL min^−1^, 200 μL) followed by the injection of the HRP-labelled secondary antibody (50 μL min^−1^, 200 μL). Amperometric measurements were then carried out at −0.1 V using TMB reagent (4 min, 50 μL min^−1^) followed by buffer injection (4 min, 100 μL min^−1^). The sensor surface regeneration was achieved with the injection of 0.1 M HCl (1 min, 100 μL min^−1^) twice for the sequential detection of different *Salmonella* concentrations. 

The gold nanoparticle-enhanced bioassays (Scheme 2) were performed using the same procedure. In this case, the HRP-labelled secondary antibody was conjugated with AuNPs prior to the experiments to amplify the sensor signal. The AuNP conjugation protocol was described in our earlier studies [18] and used as is in this work. The secondary antibody-labelled AuNPs were stored at +4 °C and warmed to room temperature before use. The concentration of nanoparticles was calculated at 525 nm wavelength and the AuNP solution was diluted based on the dilution factor calculated by considering the OD value. Commercially available *S. typhimurium* and real *S. typhimurium* samples from human stool were detected in a concentration range from 1 to 5.41 × 10^7^ cfu mL^−1^ using the amperometric sensor.

### 2.8. Development of the DNA Biosensor

#### 2.8.1. Immobilization of Neutravidin and DNA Capture Probe on the Sensor Chip Surface

The SAM formation was initially performed on the sensor chip using 2 mM MUDA as described in Section 2.4. The chip and the PMMA cassette were then assembled together using a double-sided sticky tape and docked to the sensor device that was primed with degassed Dulbecco’s modified phosphate-buffered saline (PBS, pH: 7.4). The same reagent was used as the running buffer during NA immobilization and continuously flowed over the sensor surfaces between the injections. The flow rate of the sensor was 50 μL min^−1^ during the DNA bioassays unless written otherwise. The SAM-coated electrode surfaces of the sensor chip were activated using amine coupling chemistry [29,31]. For this, a mixture of EDC (0.4 M)-NHS (0.1 M) at 1:1 volume ratio was injected to the surface during 4 min. The NA prepared in PBS (50 µg mL^−1^, 250 μL) was then immobilized to the sensor surface followed by the injection of ethanolamine (1 M, 250 µL^−1^ pH: 8.5) for blocking of the NA-free areas on the surface [31]. After immobilization of NA, the running buffer was changed to Tris buffer (20 mM Tris-HCI, 150 mM NaCl, and 1 mM EDTA, pH: 7.0) and it was used during the capturing of the biotinylated complementary surface probe and the detection of *Salmonella* DNA. The surface probe was prepared in Tris buffer in the concentration of 10 μM and injected to the NA-immobilized surface for 5 min, followed by a 1 min injection of 10 mM biotin to block the remaining active sites of the NA surface. To obtain a control surface, a biotinylated non-specific surface probe was captured on the NA layer and used for the target detection assays. The sensor signals obtained upon the binding of *Salmonella* DNA on the non-specific surfaces were subtracted from the results obtained on the target specific surfaces.

#### 2.8.2. Preparation of HRP-NA-Labelled AuNPs 

**Scheme 3:**
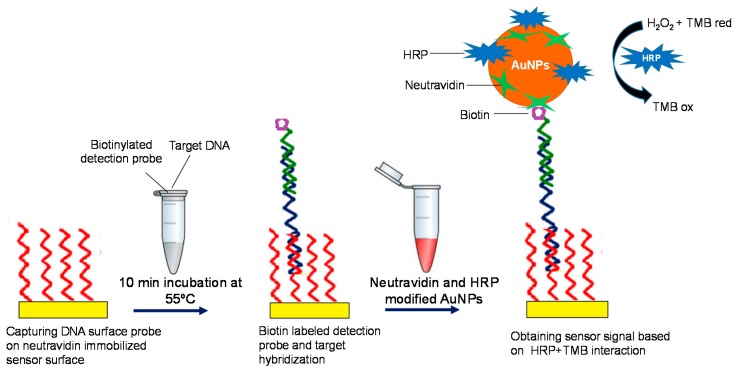
Principle of the DNA assay using the microfluidic-based electrochemical biosensor.

The HRP-NA labelled AuNPs were used for the sensitive detection of the target DNA. In this assay, HRP is required to convert TMB_red_ to TMB_ox_, and produce a measurement signal using the amperometric sensor (Scheme 3). It was well defined that proteins can physically bind to the gold surface via electrostatic interactions [32]. Herein, the same approach was used for modification of AuNPs with HRP and NA. For this, a 1 mL solution of the gold nanoparticles (15 nm) was derivatised with NA (1.5 µL, 1 mg mL^−1^) and HRP (2.5 µL, 1 mg mL^−1^) in an Eppendorf tube that was covered by aluminum foil to prevent exposure to light. This solution was incubated for 45 min on a shaker at room temperature prior to centrifugation for discarding excess reagents. The supernatant was removed, and 30 μL of BSA (10 mg mL^−1^) and 100 μL Tris buffer (20 mM) were added to the tube, respectively. The modified AuNPs were stored at +4 °C until use. The AuNP concentration was determined using a spectrophotometer at a wavelength of 525 nm. 

#### 2.8.3. DNA Detection Assay 

**Figure 2:**
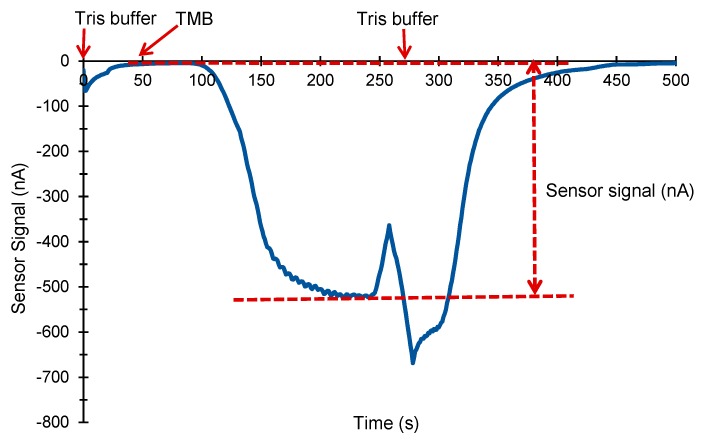
The current response of an individual electrode at −0.1 V potential is recorded uninterruptedly during the consequent injections of buffer and TMB substrate to the HRP-bound electrode to obtain a sensorgram. The flow rate of the device is 50 µL min^−1^ during the injections.

Specific gene for *Salmonella* spp. was selected based on the literature [33]. For DNA assays, the biotinylated surface probe was initially captured by the NA immobilized surface. The concentrations of capture (10 µM) and detection (10 µM) probes were kept the same for all experiments. The biotin-free *Salmonella* DNA was incubated with the biotinylated detection probe for 10 min at 55 °C using a thermal cycler. The hybridized DNA molecules were then injected to the sensor surface during 5 min (250 µL) followed by the injection of the HRP-NA modified AuNPs for 4 min (200 µL). The modified AuNPs bound to the sensor surface via interaction between the biotin label of the detection probe and the NA label of the AuNPs. The electrochemical signal was obtained using a real-time electrochemical profiling^TM^ (REP^TM^, TUBİTAK-BİLGEM, Kocaeli, Turkey) assay at a fixed potential of −0.1 V and the current was measured continuously. The current values were recorded during Tris buffer injection and this signal was considered as a baseline. The subsequent injection of 200 μL TMB revealed a current change and the subtraction between the current obtained during TMB and buffer was used as the sensor signal (Figure 2).

## 3. Results and Discussion

### 3.1. Antibody Sensor for Salmonella Detection

**Figure 3:**
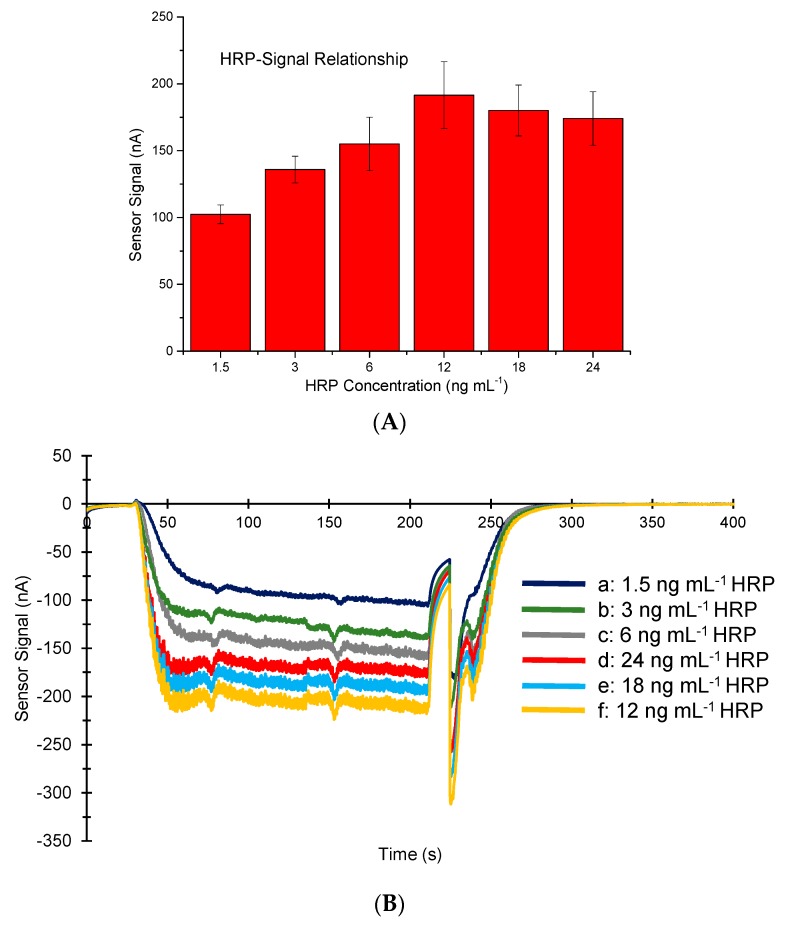
Overall results of HRP-TMB optimization assays in a concentration range of 1.5–24 ng mL^−1^ (**A**) and real-time sensorgrams obtained with six different HRP concentrations (**B**).

Prior to developing the bioassays, we initially investigated the optimum HRP concentration for use in electrochemical sensor. The sensor signal gradually increased from 1.5 ng mL^−1^ to 12 ng mL^−1^ of HRP, whereas it showed a clear decrease afterwards (Figure 3). Hence, 12 ng mL^−1^ was selected as the optimum concentration for HRP and used for the entire study. 

#### 3.1.1. Standard Sandwich Assay for the Analysis of Commercial and Real Samples

**Figure 4:**
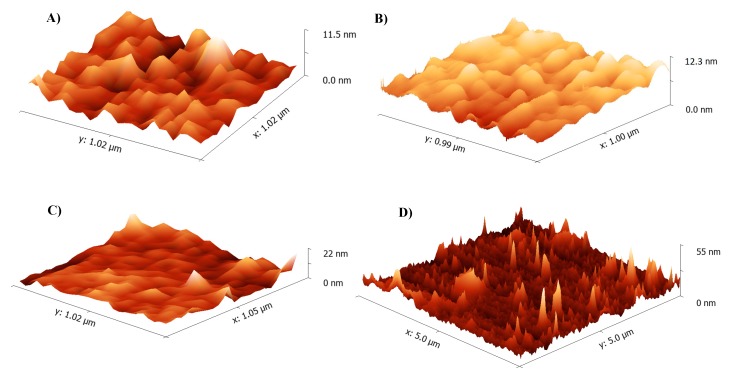
AFM analysis of bare (**A**), MUDA-coated (**B**) and antibody-immobilized (**C**,**D**) sensor surfaces.

The initial investigations were carried out with commercially available *Salmonella typhimurium* samples using a standard sandwich assay. The bare, the SAM-coated, and the antibody-immobilized sensor surfaces were characterized by AFM (Figure 4). The 3D surface topology images in 1 × 1 µm scanning area resulted in the heights of 11.5 nm, 12.3 nm, 22 nm for bare (Figure 4A), MUDA-coated (Figure 4B), and antibody-immobilized (Figure 4C) surfaces, respectively. The gradual increase of the surface height confirmed the successful preparation of the sensor chip for bacteria detection assays.

**Figure 5:**
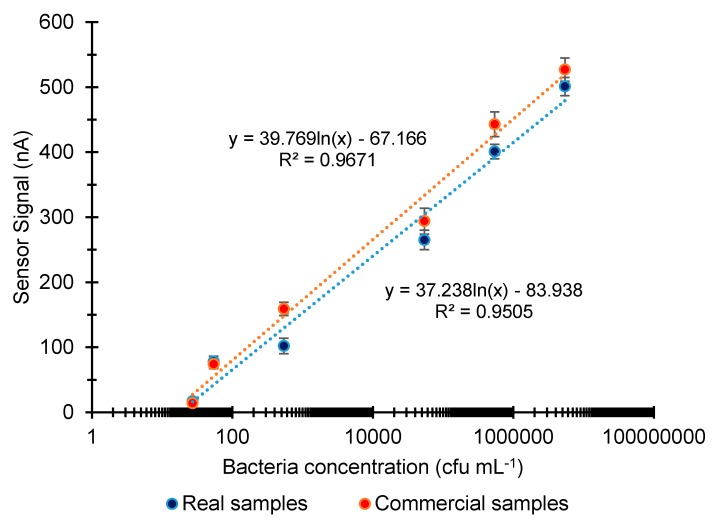
Overall results of *Salmonella* detection in a concentration range of 27–5.41 × 10^7^ using a standard sandwich assay (n = 6).

*Salmonella* detection was investigated in the concentration range of 1–5.41 × 10^7^ cfu mL^−1^, which provided a limit of detection (LOD) of 2.7 × 10^1^ cfu mL^−1^ for both commercial and real samples. The average signal ratio in the entire concentration range between commercial and real sample analyses was determined as 1.1. The overall results of *Salmonella* detection were subjected to the logarithmic regression analysis that resulted in the correlation coefficients of 0.9671 and 0.9505 for commercial and real samples, respectively (Figure 5). 

#### 3.1.2. Nanoparticle Enhanced Sandwich Assay for the Analysis of Commercial and Real Samples

**Figure 6:**
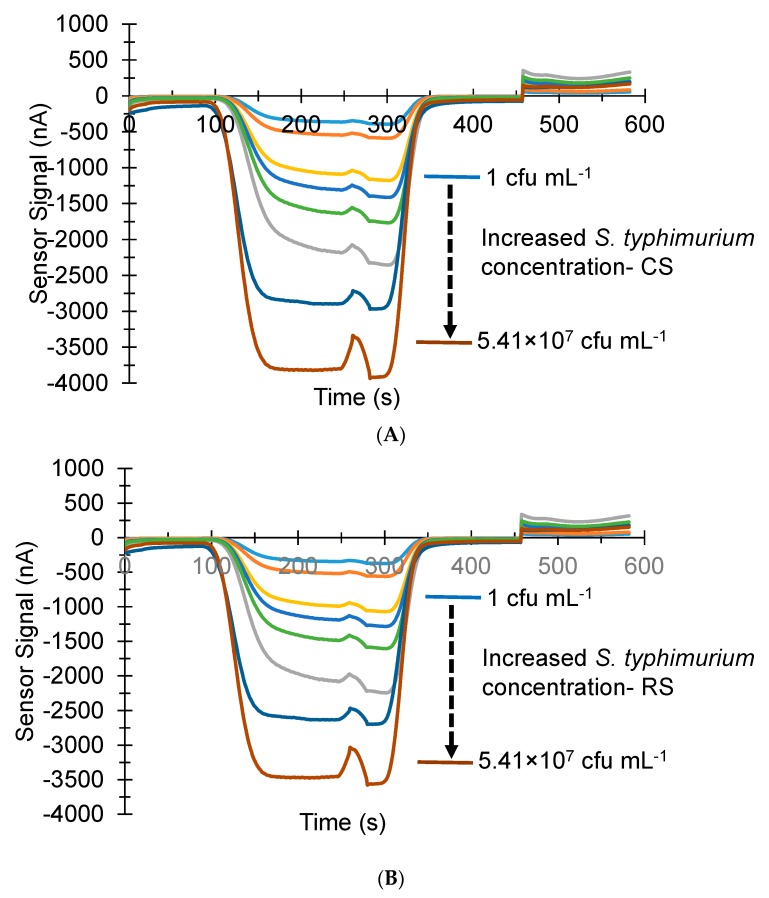

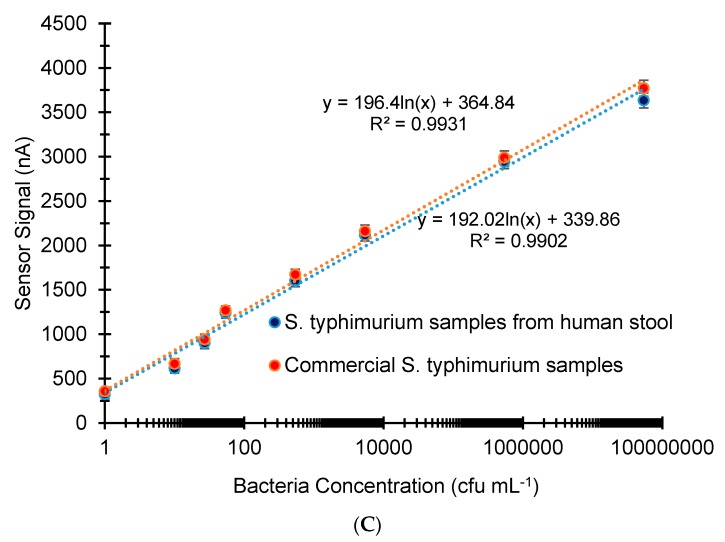
Real-time sensorgrams of commercial (**A**) and real *S. typhimurium* (**B**) detection in a concentration range of 1–5.41 *×* 10^7^ cfu mL^−1^. Overall results of the AuNP-enhanced sandwich assays for the determination of commercial and real samples (n = 6) (**C**). CS: commercial sample, RS: real sample.

The use of nanomaterials significantly enhanced the sensor signal and allows detecting trace amounts of pathogens that has critical role in diagnostic [11]. To obtain a more sensitive diagnostic assay for *Salmonella* detection we, therefore, used the AuNP-modified sandwich assay. The sensor signal obtained with AuNP conjugated detector antibody is proportional to the amount of bacteria captured on the surface by the primary antibody. The commercial and real *Salmonella* samples were investigated in a concentration range of 1–5.41 × 10^7^ cfu mL^−1^ (Figure 6A), which resulted in a 1 cfu mL^−1^ LOD (Figure 6A,B). The logarithmic regression analysis of all results revealed a good reproducibility for the assays with correlation coefficients of 0.9931 and 0.9902 for commercial and real sample analysis, respectively (Figure 6C). The signal ratio between the two assays was calculated based on the detection limit responses of two assays and it was found to be 1.098. In the entire investigation range, the average signal ratio of commercial sample analysis to real sample analysis was found to be 1.03. Therefore, for the first time in this study, we demonstrated that the sensor can reliably measure *Salmonella* samples from different sources with a very high accuracy. Additionally, 50 times higher sensitivity was obtained in this work when compared to our earlier work [18] and this is most likely due to the new chip design. 

Liebana et al. investigated *Salmonella* detection in milk using an electrochemical magneto-immunosensor where the bacteria were captured and preconcentrated from milk samples by utilizing magnetic beads via an immunological interaction [24]. A limit of detection of 7.5 × 10^3^ cfu mL^−1^ in milk diluted 1/10 in lysogeny broth was achieved in 50 min without any pretreatment. Another magneto-immunosensor for *Salmonella* quantification was reported using nano- and micro-sized magnetic particles that allowed measuring *S. enterica* down to 5 × 10^4^ and 1 × 10^4^ cfu mL^−1^, respectively [15]. A disposable immunosensor, combining a permanent magnet under the screen-printed carbon electrode and AuNPs as signal amplification agents, offers an alternative platform for sensitive detection of *Salmonella enterica* subsp. enterica serovar Typhimurium LT2 (S) [14]. This sensor was able to measure the target bacteria in a linear concentration range from 10^3^ to 10^6^ cfu mL^−1^ with a detection limit of 143 cfu mL^−1^. The AuNP enhanced biosensor technique combined with magnetic field application provided more sensitive detection platform than the conventional commercial method carried out for comparison purposes. The total sensor assay time using the disposable immunosensor was 1:30 h per sample [14]. Electrochemical sensing of *Salmonella typhimurium* in milk samples was investigated using iron oxide/gold core/shell nanomagnetic probes and CdS biolabels [21]. The screen-printed carbon electrodes were used as the sensor substrate and the measurements were carried out by square-wave anodic stripping voltammetry through the use of CdS nanocrystals. The bacterium was measured between 1 × 10^1^ and 1 × 10^6^ cfu mL^−1^, and a LOD of 13 cfu mL^−1^ was recorded. The determination of *Salmonella* in milk samples using the biosensor required less than 1 h [21]. On the other hand, the AuNP modified sandwich assay in our work could detect *S. typhimurium* in a concentration range of 1–5.41 × 10^7^ with LOD of 1 cfu mL^−1^, which demonstrates much higher sensitivity than all of the reported works. Additionally, the preparation of our antibody sensor required 30 min and the detection of a sample was achieved in only 12 min. Furthermore, the same sensor surface could be used multiple times in this work, avoiding the preparation of the antibody-immobilized surface all the time. The reliability and accuracy of the sensor are also very high, which could be demonstrated by studying with different sample sources. 

#### 3.1.3. Cross-Reactivity Studies for *Salmonella*

**Figure 7:**
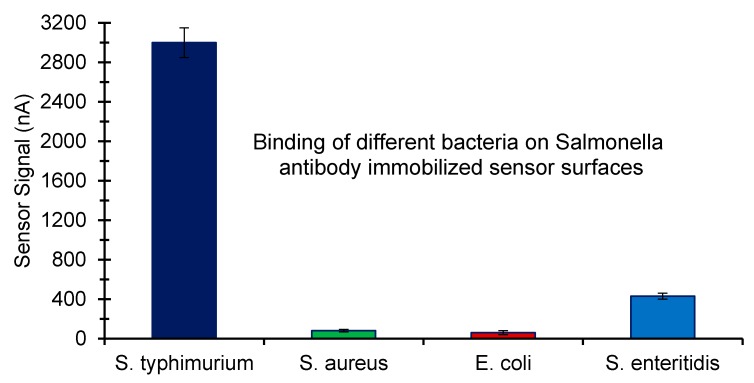
Cross-reactivity tests for *S. typhimurium* specific antibody sensor assay (n = 3).

To determine the specificity of the developed assays for the detection of *Salmonella*, the interaction of non-specific bacteria with the *Salmonella*-specific antibody-immobilized surfaces was studied. *S. typhimurium* was used as the target bacterium, whereas *S. aureus* and *E. coli* were investigated as the non-specific bacteria. Being a *Salmonella* serotype the binding of *S. enteritidis* on the antibody immobilized surface was also tested. A fixed concentration (10^7^ cfu mL^−1^) of each bacterium was used for cross-reactivity tests. The non-specific binding responses of *S. aureus* and *E. coli* were calculated as 2.66% and 2.01%, respectively, suggesting the high specificity of the bioassay for *Salmonella* (Figure 7). The binding of *S. enteritidis* on the *Salmonella* antibody immobilized surface was determined as 14.35%. Nevertheless, the specificity of antibody sensors for bacteria is often a problem, which motivated us to develop a DNA biosensor in this work as an alternative method. 

### 3.2. DNA Sensor for Salmonella Detection

**Figure 8:**
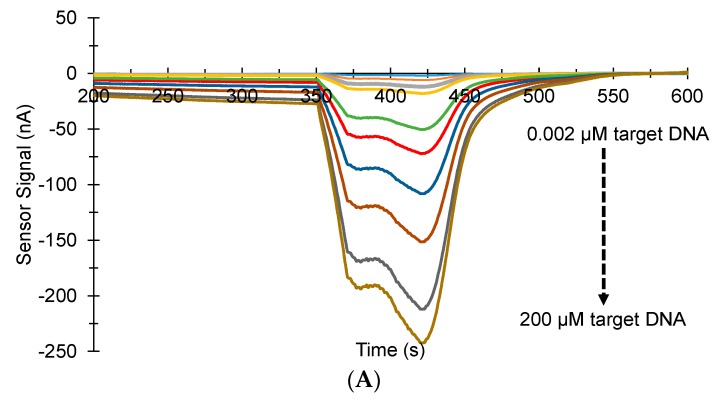

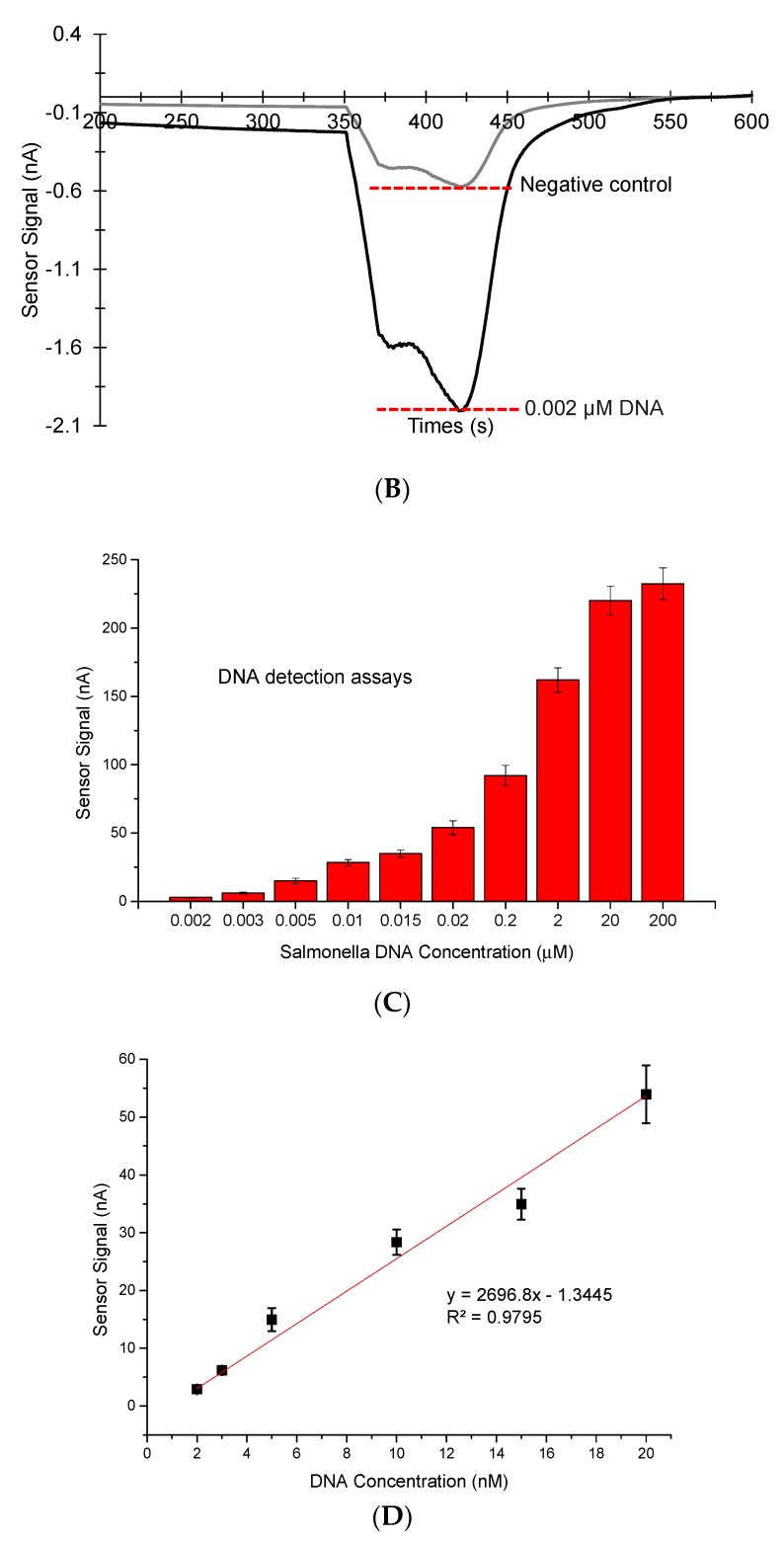
Concentration-dependent real-time sensorgram of the DNA assay (**A**). Real-time sensorgram showing signal difference between the lowest DNA concentration and negative control (**B**). The detection of *Salmonella* DNA in a concentration range of 0.002–200 µM along with standard deviations on the data (n *=* 6) (**C**). The linear calibration curve of DNA assays with a correlation coefficient of 0.9795 (**D**).

For the first time, we investigated the potential of our custom-designed MiSens device for the development of a DNA biosensor for pathogenic bacteria detection in this study. The detection of target *Salmonella* DNA was studied in a concentration range of 0.002–200 µM using the DNA surface probe that was initially captured by a neutravidin-immobilized layer on the sensor chip (Figure 8A). The sensor could differentiate the lowest concentration of DNA (0.002) and negative control (Figure 8B) and showed a good reproducibility in a wide investigation range (Figure 8C). The LOD was verified based on the linear portion of the saturation graph by calculating three times the standard deviation of the blank response (0.4 *×* 3 = 1.2 nA) and extrapolating the sensor signal in the linear calibration curve to convert the value to concentration [34]. The limit of detection was determined to be 0.94 nM with a linear range from 2 to 20 nM, as shown in Figure 8D.

**Figure 9:**
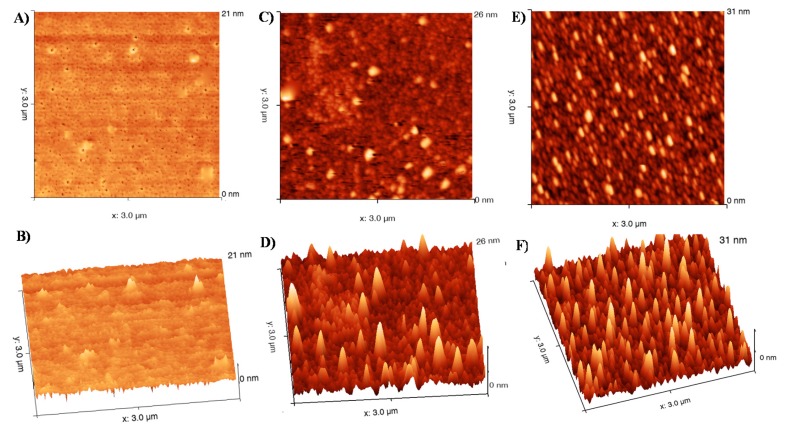
AFM 2D height images (upper part) and 3D topography images (lower part) of neutravidin immobilization (**A**,**B**), DNA surface probe capture (**C**,**D**), and target DNA binding at 2 µM concentration (**E**,**F**).

Every step of the DNA assay was characterized by employing AFM. The 2D height images and the 3D surface topography images were analyzed in the scanning area of 3 *×* 3 µm (Figure 9). A clear difference in height was observed between MUDA-coated (12.3 nm) and the NA immobilized (21 nm) sensor surfaces, indicating the effective NA immobilization for the DNA surface probe capture. When the surface probe was captured by the NA layer, a further increase in height (26 nm) was observed and the 3D surface topology image also displayed a rougher surface than that of the NA immobilization. Upon binding of the target DNA on the surface, the height reached 31 nm and the surface roughness significantly increased. 

DNA biosensors constitute strong alternatives to the antibody sensors for diagnosis of pathogenic bacteria by quantifying the genetic material of a bacterium of interest. A disposable electrochemical sensor developed for *Salmonella* detection based on a DNA hybridization reaction using the thiolated DNA capture probes could quantify *Salmonella* DNA from 5 µM to 20 µM. The hybridization event was detected using the ruthenium complex as electrochemical indicator. The sensor demonstrated high selectivity towards *Salmonella* when compared to *E. coli* [35]. The DNA sensor constructed in the current study detected *Salmonella* DNA down to 0.002 µM, which displays enormously higher sensitivity than the disposable electrochemical sensor. However, it is worth mentioning that we have not tested the sensor against other non-specific bacteria species in this work as the main focus of the current study was to investigate the potential of our custom-designed sensor for the construction of DNA assays for pathogenic bacteria detection. Instead, the specificity of the DNA assays was checked using the control surface probe and no signal was observed upon the target injection to the sensor. 

Zhang et al. used the biotinylated single-stranded DNA capture probe, immobilized onto a streptavidin-coated dextran sensor surface, to detect a specific sequence in the invA gene of *Salmonella* by employing a label-free SPR biosensor (i.e., Biacore X™, BIAcore AB). The target DNA could be measured in the range of 5‒1000 nM with a detection limit of 0.5 nM. This sensor was used for the detection of single-stranded invA amplicons from three serovars of *Salmonella*, including Typhimurium, Enterica, and Derby, and the responses to PCR products were related to different *S. typhimurium* concentrations in the range of 10^2^–10^10^ cfu mL^−1^. The hybridization reaction using the SPR sensor required 15 min, whereas the electrochemical sensor in this work allowed the DNA hybridization only in 5 min. The surface regeneration could be achieved in both studies for multiple usage of the same chip using glycine-HCI (10 mM, pH: 3.0) [36] and HCI (100 mM, pH: 3), respectively. The use of gold nanoparticles in the DNA sensor development offers superior sensitivities that play a crucial role while working with interfering media, such as milk and blood. The DNA sensors employing AuNP functionalized probes can quantify trace levels of *Salmonella* (for e.g., 6 cfu mL^−1^) in such complex sample media [20]. The AuNP-enhanced DNA biosensor in this work shows great potential for *Salmonella* detection and our future direction will be the further characterization of the developed sensor to be used in complex samples that may have a significant impact in clinical diagnosis, environmental monitoring, and food safety.

## 4. Conclusions

In this work, antibody and DNA-based biosensors were developed using a fully-automated custom-designed microfluidic device. Two different antibody biosensors were constructed using standard and nanomaterial-enhanced assay approaches. The nanomaterial-enhanced immunosensor (LOD: 1 cfu mL^−1^) increased the limit of detection around 27-fold when compared to the standard sandwich assay (LOD: 27 cfu mL^−1^) for *Salmonella* determination from human stool samples. On the other hand, the potential of the microfluidic sensor for the DNA analysis of pathogenic bacteria was successfully demonstrated for the first time. The developed DNA biosensor could detect the *Salmonella* DNA in a wide concentration range of 0.002–200 µM with a LOD of 0.94 nM. Both antibody and DNA biosensors have shown high sensitivity and specificity for *Salmonella* samples. The sensor surface could be regenerated multiple times in all assay types, which significantly reduces the cost of the system, as well as the required analysis time by skipping the receptor immobilization step. The developed sensors can be used for the diagnosis of infectious diseases caused by pathogenic bacteria.
